# Genome-Wide Characterization of the *HOX* Gene Family: Evolution and Expression Patterns in Donkey

**DOI:** 10.3390/ijms27010038

**Published:** 2025-12-19

**Authors:** Xiaotong Liu, Anqi Liu, Muhammad Zahoor Khan, Qifei Zhu, Yunfan Zheng, Wenting Chen, Bingbing Cai, Zhiyu Yan, Yongdong Peng, Changfa Wang

**Affiliations:** Liaocheng Research Institute of Donkey High-Efficiency Breeding and Ecological Feeding, College of Agriculture and Biology, Liaocheng University, Liaocheng 252000, Chinazahoorkhan@lcu.edu.cn (M.Z.K.);

**Keywords:** *HOXs*, donkey, structure, chromosome mapping, phylogenetic analysis

## Abstract

The *HOX* gene family plays an indispensable role in regulating embryonic development, cell differentiation, and morphogenesis. This study employed bioinformatics approaches for systematic analysis, ultimately identifying 33 *HOX* gene family members from the donkey genome. Physicochemical property analysis revealed that the number of amino acids encoded ranged from 94 to 444, with 31 members classified as alkaline proteins. Their secondary structure was predominantly composed of random coils and alpha helices, and all members were localized to the nucleus. Conserved motif analysis further demonstrated that all donkey HOX family proteins contained highly conserved motifs 1 and 2. Along with three other species, the 33 donkey *HOX* genes were clustered into eight phylogenetic subgroups. Furthermore, collinearity analysis indicated a high degree of collinearity between the donkey and horse *HOX* gene families. Gene Ontology analysis confirmed the significant role of the donkey *HOX* gene family in regulating embryonic development and skeletal system formation. Tissue expression profile analysis revealed significant differences in the expression levels of the 33 *HOX* genes across 13 different tissues. This study not only systematically identified and characterized the donkey *HOX* gene family but also provided valuable insights into the genetic regulation mechanisms of key traits in donkey molecular breeding.

## 1. Introduction

Homeobox genes are a class of core regulatory genes that are widely distributed across various organisms and encode transcription factors. These genes primarily regulate the expression of downstream target genes through the proteins they produce. Their products play crucial roles in processes such as embryonic development, cell differentiation, and morphogenesis by controlling the spatiotemporal expression of these target genes [1,2]. The *HOX* gene family is characterized by several distinctive features, including high conservation, clustered organization, and spatiotemporal collinearity. High conservation is exemplified by a shared 180 bp highly conserved DNA sequence (the homeobox) that encodes a protein domain known as the homeodomain, which consists of 60 amino acids [3,4]. The *HOX* family is extensive and diverse, typically organized into four distinct clusters—*HOXA*, *HOXB*, *HOXC*, and *HOXD*—each located on different chromosomes. Additionally, *HOX* gene clusters exhibit a high degree of organizational coherence, with all genes transcribed from the same DNA strand [3,5]. *HOX* expression demonstrates collinearity, meaning that genes within the cluster activate sequentially along the 3′→5′ direction of the chromosome. The expression regions of *HOX* genes are strictly partitioned along the anterior–posterior axis of the embryo: genes located at the 3′-end are expressed in the anterior region, while those at the 5′-end are expressed in the posterior region, with intermediate genes expressed in the transitional zone between them. Furthermore, within the same axial region, if both 3′-end and 5′-end *HOX* genes are expressed simultaneously on a single chromosome, the 5′-end genes exhibit posterior prevalence [2,6].

*HOX* genes are crucial regulatory elements that govern vertebral patterning in vertebrates. Mutations in these genes can lead to variations in vertebral morphology and number. Specifically, the loss of function in *HOXC8* and *HOXD8* primarily disrupts the patterning of the lower thoracic and lumbar vertebrae, while the loss of *HOXB8* affects the cervicothoracic region. The simultaneous knockout of two or three *HOX* genes results in more severe and widespread defects [7]. Ectopic expression of *HOXC8* in mice induces skeletal abnormalities; gain-of-function mutations in this gene can transform the first lumbar vertebra into an additional thoracic vertebra, complete with a corresponding pair of ribs [7]. In mouse models where all members of the *HOX10* or *HOX11* gene families are knocked out, the complete loss of *HOX10* function inhibits the formation of lumbar vertebral bodies. In the absence of *HOX11* function, the sacral vertebrae fail to develop normally, with vertebrae destined to form the sacrum instead exhibiting morphological characteristics typical of lumbar vertebrae [8]. Observations of *HOXD4* mutant mice reveal distinctive skeletal abnormalities in both heterozygous and homozygous individuals, specifically manifested as the homeotic transformation of the second cervical vertebra (C2) into the first cervical vertebra (C1), accompanied by malformations of the neural arches and basioccipital bones in the first three cervical vertebrae (C1–C3) [9]. Additionally, mice with mutations in the *HOX5*, *HOX6*, and *HOX9* genes also display abnormalities in the ribs and sternum [10].

The molecular mechanisms by which *HOX* genes regulate vertebral development provide a compelling genetic framework for understanding economically important skeletal traits in livestock species, including donkeys. Given the documented effects of *HOX* gene mutations on vertebral number and morphology in model organisms, these genes represent promising candidates for genetic improvement of body conformation traits in donkey breeding.

China has a long-standing tradition of consuming donkey meat, which is characterized by high protein content, a rich profile of essential amino acids, high levels of unsaturated fatty acids, and low fat, cholesterol, and calories, making it a high-quality meat source [11]. Additionally, donkey-hide gelatin (ejiao) has been a vital component of traditional Chinese medicine, used for millennia as a valuable herbal remedy known for its blood-nourishing and stamina-enhancing properties [12]. With the growth of the health industry, the market demand for donkey meat, milk, and related products continues to expand. However, the inherent reproductive characteristics of donkeys, such as single births and long generation intervals, severely constrain industrial supply. Consequently, enhancing individual production performance through genetic improvement has emerged as a critical developmental strategy. Vertebral number is a significant economic trait in livestock, with variations that can substantially impact body conformation, hide yield, wool production, and meat production. Existing research indicates that an additional vertebra in Dezhou donkeys correlates with positive increases in both body length and carcass weight. Statistical analysis reveals that an extra thoracic vertebra in Dezhou donkeys increases average body length by 4.3 cm, while an additional lumbar vertebra contributes an increase of 2.4 cm in average body length. Each additional vertebra is associated with an average live weight gain of 7.2 kg and a net hide weight gain of 0.65 kg [13], highlighting its significant breeding value and economic returns.

Given the pivotal role of the *HOX* gene family in morphogenesis and vertebral development, this study utilizes donkeys as the model organism. Subsequent analyses included chromosomal localization, physicochemical properties, secondary structure prediction, subcellular localization, gene structure, conserved motifs, phylogenetic analysis, interspecies collinearity assessment, Ka/Ks analysis, expression patterns, and functional enrichment. Therefore, this study aims to identify and characterize members of the donkey *HOX* gene family through the bioinformatics methods and establish a foundation for further investigation into the functional and regulatory mechanisms of the donkey *HOX* gene family.

## 2. Results

### 2.1. Identification of Members of the Donkey HOX Gene Family

**Figure 1:**
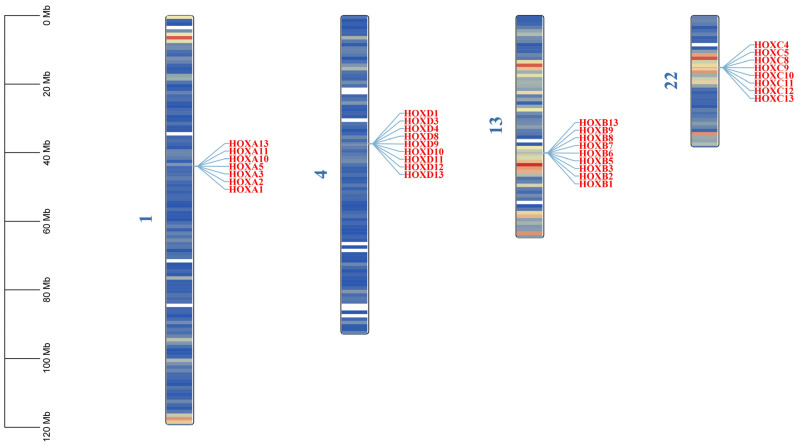
Chromosomal location of *HOX* gene family in donkeys. The color on the chromosome represents the size of gene density in the 1 MB window, blue represents the region with low gene density, and red is the opposite.

Through HMMER (v3.3.2+dfsg-1)and TBtools Ⅱ-BLAST (v2.340) searches, combined with conserved domain validation using CDD and SMART databases, 33 *HOX* gene family members were identified in the donkey genome. These genes range in length from 958 to 24,874 bp. Sixteen genes are located on the forward strand, while 17 are on the reverse strand (Table 1). The *HOX* genes are distributed across four chromosomes: 1, 4, 13, and 22 (Figure 1). The number of *HOX* genes in donkeys (33) is lower than in humans (39), horses (35), and cattle (39), with the primary missing genes being *HOXA4*, *HOXA6*, *HOXA7*, *HOXA9*, *HOXB4*, and *HOXC6* (Table 2).

### 2.2. Analysis of the Basic Properties of Proteins from the Donkey HOX Gene Family

Analysis of physicochemical properties revealed that the amino acid length of donkey HOX family members ranged from 94 (HOXA10) to 444 (HOXA3) (Table 3). Protein molecular weights varied between 11,452.25 Da (HOXA10) and 47,378.77 Da (HOXA3). The theoretical isoelectric point (pI) ranged from 5.04 (HOXB2) to 10.76 (HOXD8), with 31 members classified as alkaline proteins (pI > 7). The instability index ranged from 32.48 (HOXA10) to 90.52 (HOXB2). The aliphatic index ranged from 41.74 (HOXD9) to 73.59 (HOXD12). The grand average of hydropathicity (GRAVY) ranged from −1.518 (HOXD8) to −0.288 (HOXA13). All GRAVY values were negative, indicating that all HOX proteins are hydrophilic.

Secondary structure prediction revealed that donkey HOX proteins predominantly contain random coils, followed by α-helices, with β-turns representing the smallest proportion. Subcellular localization analysis showed that all donkey HOX proteins are localized to the nucleus (Table 4).

### 2.3. Analysis of Gene Structure and Conserved Motifs in the Donkey HOX Gene Family

**Figure 2:**
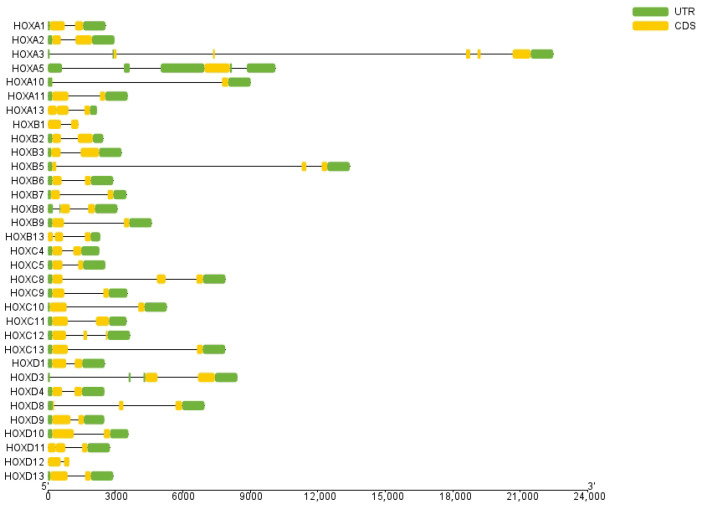
Structure analysis of *HOX* gene family members in donkey.

**Figure 3:**
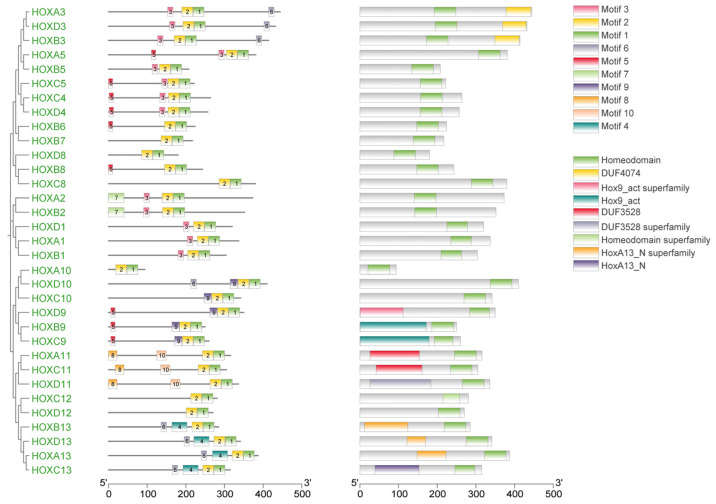
Phylogenetic tree, motif and domain relationship of donkey *HOX* gene family.

Gene structure analysis revealed that exon numbers in donkey *HOX* gene family members ranged from 1 to 5 (Figure 2). *HOXA5* contained the fewest exons (1), while *HOXA3* contained the most (5). All genes except *HOXA5* contained introns, with *HOXA3* having the highest number. These results indicate structural diversity among donkey *HOX* family members, reflecting evolutionary divergence within this gene family. Conserved motif analysis identified ten distinct motifs in the donkey *HOX* protein sequences (Figure 3). Motifs 1 and 2 were present in all 33 *HOX* family members, indicating high conservation of these sequences. The remaining motifs showed variable distribution patterns across different *HOX* members. This conservation pattern suggests that motifs 1 and 2 likely represent core functional domains essential for HOX protein function. Analysis results from the PFAM (PF00046) and CDD databases confirmed that both motif 1 and motif 2 are located within the homeodomain of the HOX protein. Functional analysis demonstrates that motif 1 possesses DNA-binding capability and DNA-binding transcription factor activity; motif 2 has also been confirmed to participate in the DNA-binding process.

### 2.4. Interspecies Phylogenetic Tree Analysis of the HOX Gene Family

**Figure 4:**
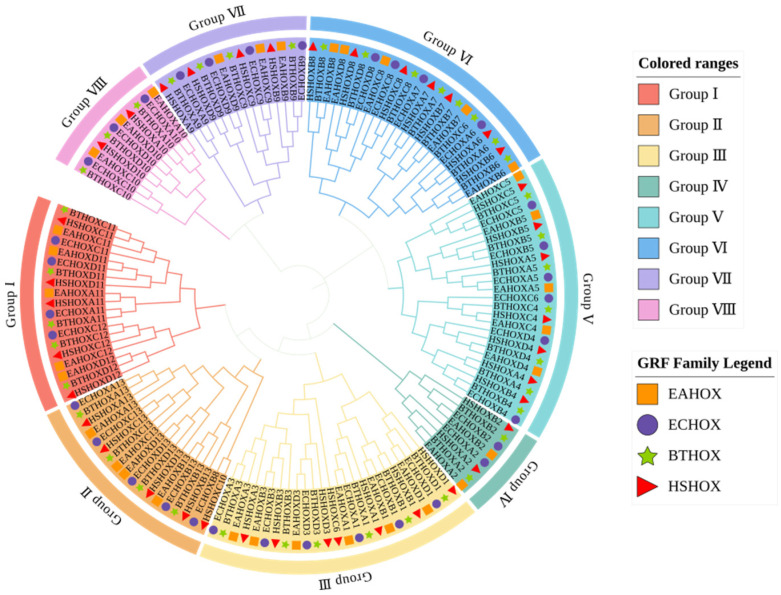
Phylogenetic tree among species of *HOX* gene family.

To investigate the evolutionary relationships among donkey *HOX* gene family members and those of other species, phylogenetic analysis was performed using HOX protein sequences from four species: donkey, horse, cattle, and human. A phylogenetic tree was constructed using the neighbor-joining (NJ) method in MEGA software (v12.0.11) (Figure 4, Supplementary Materials S2). The dataset comprised 145 HOX proteins: 33 from donkey, 34 from horse, 39 from cattle, and 39 from human.

The phylogenetic tree clustered 145 *HOX* genes into eight subfamilies (Group I to Group VIII). *HOX* gene family members from the four species clustered together within each subfamily, indicating close evolutionary relationships and potential functional conservation. Each subfamily contained orthologous gene pairs between donkey and horse. Group II contained the fewest orthologous pairs (one pair: *HOXD13*), while Group III contained the most (five pairs: *HOXB3*, *HOXD3*, *HOXA1*, *HOXB1*, and *HOXD1*). Groups IV and VII each contained two pairs, while Groups I, V, VI, and VIII each contained three pairs.

### 2.5. Interspecies Collinearity Analysis

**Figure 5:**
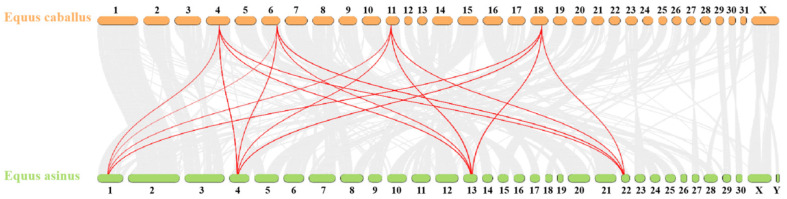
Collinear analysis of *HOX* gene family in Equus asinus and Equus caballus. The gray line represents all collinearity blocks of the whole genome; The red line represents the collinearity of *HOX* gene family.

To further elucidate the evolutionary relationships within the donkey *HOX* gene family, an interspecies collinearity analysis was conducted on the *HOX* gene families of donkeys and horses. As shown in Figure 5 and Table 5, 55 pairs of homologous genes were identified between donkeys and horses, exhibiting high collinearity. This indicates a close phylogenetic relationship between the two species.

### 2.6. Ka/Ks Analysis

To assess selective pressure on the donkey *HOX* gene family, the ratio of non-synonymous (Ka) to synonymous (Ks) substitution rates was calculated for all gene pairs. The Ka/Ks ratio indicates the mode of selection: Ka/Ks < 1 suggests purifying selection, Ka/Ks = 1 indicates neutral evolution, and Ka/Ks > 1 indicates positive selection. Analysis revealed that all Ka/Ks ratios among donkey *HOX* gene family members were less than 1 (Table 6), indicating that this gene family has evolved under purifying selection. This selective constraint suggests strong functional conservation of *HOX* genes, consistent with their essential roles in development.

### 2.7. Functional Enrichment Analysis of the Donkey HOX Gene Family

**Figure 6:**
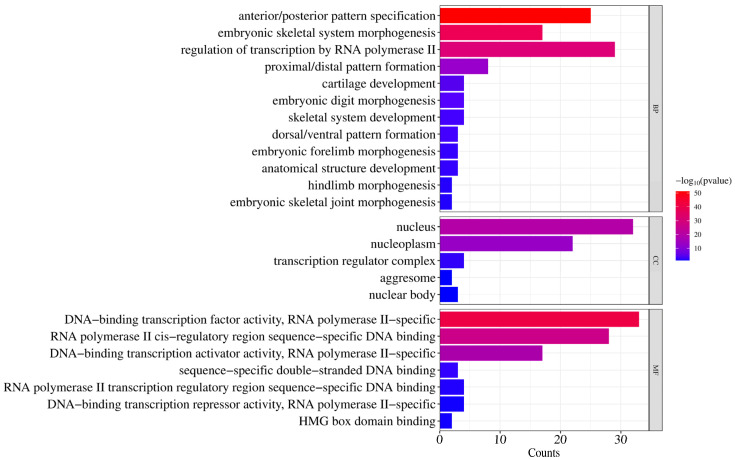
GO enrichment analysis of donkey *HOX* family members.

To elucidate the biological functions of donkey *HOX* genes, GO enrichment analysis was performed across three categories: biological processes, cellular components, and molecular functions (Figure 6).

For biological processes, significantly enriched terms included anterior/posterior pattern specification (GO:0009952), embryonic skeletal system morphogenesis (GO:0048704), proximal/distal pattern formation (GO:0009954), and cartilage development (GO:0051216). These terms are associated with skeletal system development and body axis patterning during embryogenesis. Additionally, regulation of transcription by RNA polymerase II (GO:0006357) was significantly enriched, indicating transcriptional regulatory functions.

For cellular components, significant enrichment was observed for nucleus (GO:0005634), nucleoplasm (GO:0005654), transcription regulator complex (GO:0005667), nuclear body (GO:0016604), and aggresome (GO:0016235). These results confirm nuclear localization of HOX proteins, consistent with their roles in transcriptional regulation.

For molecular functions, enrichment was primarily observed in DNA−binding transcription factor activity, RNA polymerase II−specific (GO:0000981), RNA polymerase II cis−regulatory region sequence−specific DNA binding (GO:0000978), and DNA-binding transcription activator activity, RNA polymerase II−specific (GO:0001227). These findings confirm that HOX proteins function as sequence−specific transcription factors.

### 2.8. Expression Analysis of Members of the Donkey HOX Gene Family

**Figure 7:**
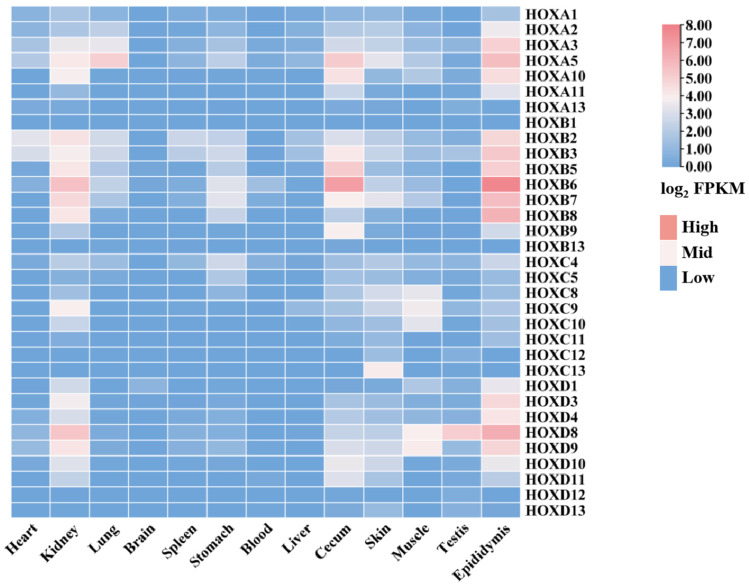
Expression of donkey *HOX* family members in different tissues.

To investigate the expression patterns of the *HOX* gene family across different donkey tissues, transcriptomic data from 13 tissues were analyzed using RNA-seq data (Supplementary Materials S1) obtained from the NCBI database. This analysis revealed distinct tissue-specific expression profiles for *HOX* family members (Figure 7, Supplementary Materials S1).

Notable expression patterns included the following: *HOXB2* and *HOXB3* showed high expression in heart, spleen, and liver. *HOXB6* and *HOXB7* were highly expressed in kidney and stomach. *HOXA3* exhibited the highest expression in lung. Most *HOX* family members showed low expression levels (FPKM < 1) in brain. *HOXB6* showed the highest expression in blood, cecum, and epididymis. *HOXC13*, *HOXA5*, and *HOXB7* were highly expressed in skin. *HOXC8*, *HOXC9*, *HOXC10*, *HOXD8*, and *HOXD9* showed high expression in muscle. *HOXD8* exhibited the highest expression in testis.

These tissue-specific expression patterns suggest that *HOX* genes may play differentiated regulatory roles in tissue development and functional maintenance. These findings provide a foundation for further investigation into the functions and regulatory mechanisms of the donkey *HOX* gene family.

## 3. Discussion

In this study, 33 *HOX* genes were identified in the donkey genome, representing a reduction in six members compared to the 39 *HOX* genes found in humans. This difference likely reflects species-specific gene loss events, variations in genome assembly quality, or lineage-specific evolutionary pressures, consistent with observations in other mammalian lineages [14,15]. The donkey *HOX* genes are organized into four clusters distributed across chromosomes 1, 4, 13, and 22, supporting the canonical vertebrate *HOX* cluster organization that originated from two rounds of whole-genome duplication during early vertebrate evolution [16,17]. This clustered arrangement is evolutionarily conserved and functionally significant, as it facilitates coordinated transcriptional regulation through shared chromatin domains [18]. In this study, all four *HOX* gene clusters exhibited a 3′→5′ linear arrangement on the chromosomes. During embryonic development, the genes within the *HOX* clusters were sequentially activated and expressed in a 3′-to-5′ direction. Specifically, the genes located at the 3′ end were predominantly expressed in the anterior tissues of the embryo (e.g., head and neck), whereas the genes proximal to the 5′ end were concentrated in the posterior tissues (e.g., lumbar and caudal). These expression patterns are consistent with the spatial collinearity characteristics of the *HOX* gene family.

Physicochemical analysis revealed that donkey HOX proteins are predominantly alkaline (31 of 33 members) and uniformly hydrophilic, with molecular weights ranging from 11.5 to 47.4 kDa [19]. These properties are consistent with nuclear localization and DNA-binding function. Secondary structure prediction showed high proportions of random coils and α-helices, with random coils likely serving as flexible linkers between structured domains [20]. Intrinsically disordered regions are known to enhance protein-protein and protein-DNA interactions through conformational plasticity [21], which may contribute to the sequence-specific DNA-binding capabilities of *HOX* transcription factors. Indeed, subcellular localization analysis confirmed nuclear localization for all 33 HOX proteins, consistent with their role as transcription factors that must access nuclear to regulate target gene expression [22,23].

Gene structure analysis revealed that most donkey *HOX* genes contain 2–3 exons and 1–2 introns, with *HOXA3* exhibiting the most complex structure. Conserved motif analysis identified ten distinct motifs across the *HOX* family, with motifs 1 and 2 universally present in all 33 members, indicating strong functional constraint on these core domains. Moreover, as a highly conserved class of transcription factors, HOX proteins play crucial regulatory roles in key processes such as somite patterning and organogenesis during animal embryonic development. The functional significance of these roles may have driven the evolutionary conservation of these motifs. Notably, paralogous groups showed consistent motif compositions; for example, *HOXA3/HOXB3/HOXD3* and *HOXA13/HOXB13/HOXC13/HOXD13* exhibited similar motif arrangements. This conservation pattern suggests functional similarity within paralogous groups and likely reflects purifying selection to maintain essential developmental functions. Structural variation among *HOX* family members has been attributed to evolutionary events including duplications, insertions, and deletions [24,25], representing a balance between functional constraint and adaptive diversification.

To further understand the evolutionary relationships within this gene family, phylogenetic analysis of HOX proteins from donkey, horse, cattle, and human was performed. The results revealed that orthologous genes clustered together across species, indicating that inter-species conservation of individual *HOX* genes exceeds intra-family conservation within a single species. This pattern is characteristic of genes under strong purifying selection to maintain conserved developmental functions. Collinearity analysis between donkey and horse genomes showed high synteny for *HOX* clusters, although their chromosomal positions differed substantially (chromosomes 1, 4, 13, 22 in donkey versus 4, 6, 11, 18 in horse). These chromosomal rearrangements [26] likely occurred during equid divergence, yet the preservation of cluster integrity underscores the functional importance of maintaining *HOX* gene linkage for coordinated transcriptional regulation.

GO enrichment analysis provided functional insights into the biological roles of donkey *HOX* genes. Significant overrepresentation was observed for biological processes including anterior–posterior pattern specification, embryonic skeletal system morphogenesis, and transcriptional regulation by RNA polymerase II. These findings align with established *HOX* functions in body axis patterning, limb development, and skeletal formation [3,27], confirming the evolutionary conservation of *HOX* gene roles across mammals. Specifically, the two SNPs g.15179224C>T and g.15179674G>A in *HOXC8* gene were significantly correlated with carcass weight, lumbar vertebrae length, and lumbar vertebrae number in Dezhou donkeys [28]. This indicates that *HOXC8* gene is a key gene influencing the number of vertebrae and body weight in the Dezhou donkey, and it may serve as a potential genetic marker for selecting and breeding high-quality, high-yielding Dezhou donkey strains. The enrichment of transcription factor activities at the molecular function level substantiates the regulatory nature of HOX proteins. These results provide a functional framework for investigating *HOX* gene contributions to economically important traits in donkeys, particularly vertebral number variation, which directly impacts body conformation, carcass weight, and hide yield [13].

Among the *HOX* genes identified in donkeys, several paralogs merit particular attention for their established roles in vertebral patterning. *HOXC8*, located on chromosome 22, is a key regulator of thoracolumbar vertebral identity; loss-of-function mutations in mice cause homeotic transformations of lumbar vertebrae toward thoracic identity, while gain-of-function mutations can induce the formation of supernumerary ribs. The association of *HOXC8* polymorphisms with vertebral number variation in Dezhou donkeys suggests this gene as a primary target for marker-assisted selection. Similarly, *HOXD4* plays a critical role in cervical vertebral specification, with mutations causing anterior transformations of the axis (C2) into atlas-like (C1) morphology. The *HOX10* paralogs (*HOXA10*, *HOXC10*, *HOXD10*) collectively specify lumbar vertebral identity; complete loss of *HOX10* function abolishes lumbar vertebral formation entirely. The *HOX11* paralogs (*HOXA11*, *HOXC11*, *HOXD11*) are essential for sacral vertebral development, with their loss causing sacral vertebrae to adopt lumbar-like characteristics. These paralog-specific functions provide a molecular basis for understanding natural variation in vertebral formulas across donkey populations and offer specific genetic targets for breeding programs aimed at optimizing body conformation.

Tissue expression profiling revealed distinct spatial patterns consistent with known *HOX* functions. The near-absence of *HOX* expression in brain tissue (FPKM < 1 for most genes) reflects the anterior expression boundary that typically excludes forebrain regions, a pattern essential for proper brain regionalization [29]. In contrast, robust expression of multiple *HOX* genes in kidney indicates active roles in organogenesis and tissue homeostasis [30]. Several expression patterns provide insights into tissue-specific *HOX* functions. In skin, 30 *HOX* genes were expressed, further confirming the role of *HOX* genes in skin development, homeostasis maintenance, and regeneration [31], with *HOXC13* showing the highest levels, consistent with its established role in hair follicle development and epithelial differentiation [32]. Donkey hide is the primary raw material for producing Ejiao (donkey-hide gelatin), with collagen as its main component [33]. The expression level of *HOXC13* may regulate the local collagen yield and the compactness of fiber arrangement by affecting hair follicle density and growth status, thereby enhancing the gelatin production rate. *HOXC13* is expected to serve as a potential molecular marker for evaluating the collagen quality of donkey hide, which provides a theoretical basis for breeding skin-purpose donkey strains that are more suitable for producing high-quality gelatin. In muscle tissue, *HOXD9* showed peak expression, suggesting involvement in myogenesis and skeletal patterning [34,35]. Furthermore, previous studies have shown that homeobox transcription factors may play a crucial role in skeletal muscle by regulating muscle fiber types and intramuscular fat [36,37], thereby influencing meat yield and quality in livestock.

The hematopoietic system showed predominant *HOXB6* expression; the dysregulation of this gene has been implicated in acute myeloid leukemia through aberrant differentiation of hematopoietic stem cells [38]. In reproductive tissues, *HOXD8* was highly expressed in testis, while 26 genes showed active expression (FPKM > 1) in epididymis, indicating potential roles in male fertility through regulation of epididymal function [39,40,41]. Finally, *HOXA5* showed elevated expression in lung tissue, consistent with its essential function in respiratory tract morphogenesis; *HOXA5* knockout mice exhibit severe postnatal lung defects including alveolar damage and emphysema [42].

This comprehensive characterization establishes a foundation for understanding *HOX* gene functions in donkey development and physiology. The tissue expression patterns, combined with functional enrichment data, suggest that specific *HOX* genes regulate vertebral patterning—a trait with significant economic value affecting body length (additional thoracic vertebra: +4.3 cm; additional lumbar vertebra: +2.4 cm), carcass weight (+7.2 kg per vertebra), and hide yield (+0.65 kg per vertebra) [13]. The conservation of *HOX* gene function across mammals indicates that insights from model organisms can inform donkey breeding strategies. Future functional studies using genome editing and chromatin profiling will be necessary to validate the regulatory roles of specific *HOX* genes in vertebral number determination and to develop molecular markers for selection of economically valuable traits in donkey production.

## 4. Materials and Methods

### 4.1. Data Preparation and Gene Family Member Identification

Initially, the complete genome sequence, gene annotation file, and protein sequence file of the donkey were obtained from the Ensembl database (https://asia.ensembl.org/info/data/ftp/index.html (accessed on 22 September 2025)). Subsequently, the Hidden Markov Model (HMM) profile (PF00046) for the *HOX* gene family was downloaded from the Pfam database. The hmmsearch program, part of the HMMER software (v3.3.2+dfsg-1, Howard Hughes Medical Institute, Ashburn, VA, USA) suite, was employed to analyze the donkey protein sequence file, facilitating the identification of *HOX* gene family members. Following this, the BLAST function within TBtools-II (v2.340, CJ-Chen Lab, Guangzhou, China) was utilized to conduct alignment between donkey protein sequences and horse *HOX* family protein sequences, which yielded potential candidate protein sequences for the donkey *HOX* family. The e-value for both HMMER and BLAST searches was set to 1 × 10^−5^. The intersection of results from both methodologies was taken to refine candidate members of the *HOX* gene family. The candidate gene family protein sequences were submitted to the CDD and SMART databases for conserved domain validation. Sequences lacking the *HOX* domain were removed to obtain the final gene family members. Gene annotation information corresponding to the *HOX* gene family member IDs was extracted from the donkey GTF gene annotation file. TBtools-II software (v2.340) was used to generate the chromosomal localization map of the donkey *HOX* genes.

### 4.2. Physicochemical Properties of Proteins, Secondary Structure Prediction, and Subcellular Localization Analysis

The physicochemical properties of donkey *HOX* gene family members were analyzed using the Protein Parameter Calculator feature in TBtools-II software, including amino acid residues, molecular weight, theoretical isoelectric point, instability index, aliphatic index, and grand average of hydropathicity (GRAVY). The secondary structure of *HOX* proteins was predicted using the SOPMA online tool (https://npsa.lyon.inserm.fr/cgi-bin/npsa_automat.pl?page=/NPSA/npsa_sopma.html (accessed on 24 September 2025)). Subcellular localization analysis of *HOX* proteins was performed using the WoLF PSORT online tool (https://wolfpsort.hgc.jp/ (accessed on 24 September 2025)).

### 4.3. Gene Structure and Conserved Motif Analysis

Gene structures were visualized using the GXF Rename and Visualize Gene Structure functions in TBtools-II software. Predictions on protein sequence files of the *HOX* gene family were performed using NCBI Batch CD-Search (https://www.ncbi.nlm.nih.gov/Structure/bwrpsb/bwrpsb.cgi (accessed on 27 September 2025)) to obtain hitdata files. Subsequently, the Simple MEME Wrapper function in TBtools-II was employed to perform conserved motif analysis on the protein sequences of the donkey *HOX* gene family, with the motif count set to 10. Finally, the Gene Structure View function in TBtools-II was used to visualize the results.

### 4.4. Phylogenetic Tree Construction

To investigate the evolutionary relationships among members of the *HOX* gene family, multiple sequence alignments of *HOX* protein sequences from donkeys, horses, cattle, and humans were performed using MEGA (v12.0.11, Institute for Genomics and Evolutionary Medicine, Temple University, Philadelphia, PA, USA) software. A phylogenetic tree was constructed using the neighbor-joining method, with the bootstrap value set to 1000, the substitution model set to p-distance and other parameters left at default settings. The resulting tree was then visualized using the online tool iTOL (https://itol.embl.de/ (accessed on 30 September 2025)).

### 4.5. Interspecies Collinearity Analysis

Based on the gene sequence files and gene annotation files of donkeys and horses, interspecies collinearity analysis was performed using the One Step MCScanX-Super Fast function in TBtools-II. Subsequently, visualization was achieved through the Dual Synteny Plot for MCScanX function within TBtools-II.

### 4.6. Ka/Ks Analysis

Intraspecific collinearity analysis was performed using the One Step MCScanX-Super Fast feature in TBtools-II to obtain gene pair files, then the Ka/Ks ratio was calculated using the Simple Ka/Ks Calculator (NG) feature in TBtools-II.

### 4.7. Functional Enrichment Analysis

Using the online tool DAVID (https://davidbioinformatics.nih.gov/ (accessed on 30 September 2025)), gene functional enrichment analysis was performed on the *HOX* gene family across three categories: biological process, molecular function, and cellular component.

### 4.8. Tissue Expression Analysis

Raw RNA-seq sequencing data (PRJNA431818) from 13 tissues (brain, heart, kidney, liver, lung, muscle, skin, spleen, stomach, blood, cecum, epididymis, and testis) of adult domestic donkeys were downloaded from the SRA database on the NCBI website (https://www.ncbi.nlm.nih.gov). Quality control was performed using FastQC (v0.11.8; http://www.bioinformatics.babraham.ac.uk/projects/fastqc/ (accessed on 30 September 2025)) followed by alignment to the donkey genome (ASM1607732v2) via HISAT2 (v2.1.0, Center for Computational Biology, Johns Hopkins University, Baltimore, MD, USA). Transcripts were assembled using StringTie, and gene expression abundance (FPKM) was estimated. Finally, the HeatMap function in TBtools-II was used to construct an expression heatmap of *HOX* family genes across different donkey tissues.

## 5. Conclusions

In this study, 33 *HOX* gene family members were identified in the donkey genome and were characterized by their chromosomal distribution, physicochemical properties, and structural features. All HOX proteins were predicted to localize to the nucleus and contained highly conserved motifs, consistent with their function as transcription factors. Phylogenetic analysis classified these genes into eight subfamilies, with strong conservation of orthologous relationships across mammalian species. Collinearity analysis revealed high synteny between donkey and horse *HOX* clusters, indicating close evolutionary relationships between equid species. Ka/Ks analysis confirmed that purifying selection maintains functional constraint across all *HOX* family members.

Functional enrichment analysis demonstrated significant roles in anterior–posterior patterning, skeletal morphogenesis, and transcriptional regulation, consistent with established HOX functions in developmental processes. Tissue expression profiling revealed distinct spatial patterns, with tissue-specific expression of particular HOX providing essential genomic resources for future research on donkey HOX genes and their roles in economically important traits.

## Figures and Tables

**Table 1:** Basic information of *HOX* gene family in donkeys.

Gene ID	Genes	Chromosomes	Start/bp	End/bp	Length/bp	Strand	Number of Exons	Number of Introns
ENSEASG00005030120	*HOXA1*	1	43,980,185	43,982,855	2671	+	2	1
ENSEASG00005033684	*HOXA2*	1	43,973,653	43,976,618	2966	+	2	1
ENSEASG00005035791	*HOXA3*	1	43,946,971	43,969,443	22,473	+	5	4
ENSEASG00005034554	*HOXA5*	1	43,924,135	43,936,004	11,870	+	1	0
ENSEASG00005022176	*HOXA10*	1	43,897,015	43,906,034	9020	+	2	1
ENSEASG00005022180	*HOXA11*	1	43,891,413	43,895,081	3669	+	2	1
ENSEASG00005022194	*HOXA13*	1	43,876,759	43,878,943	2185	+	3	2
ENSEASG00005000594	*HOXB1*	13	40,157,242	40,158,603	1362	+	2	1
ENSEASG00005000585	*HOXB2*	13	40,145,586	40,148,051	2466	+	2	1
ENSEASG00005000581	*HOXB3*	13	40,116,481	40,141,354	24,874	+	2	1
ENSEASG00005000563	*HOXB5*	13	40,086,273	40,099,698	13,426	+	3	2
ENSEASG00005000560	*HOXB6*	13	40,092,674	40,095,582	2909	+	2	1
ENSEASG00005000556	*HOXB7*	13	40,080,454	40,083,949	3496	+	2	1
ENSEASG00005024978	*HOXB8*	13	40,076,246	40,079,340	3095	+	2	1
ENSEASG00005000527	*HOXB9*	13	40,064,954	40,069,573	4620	+	2	1
ENSEASG00005025083	*HOXB13*	13	39,998,026	40,000,358	2333	+	4	3
ENSEASG00005010161	*HOXC4*	22	15,135,188	15,137,482	2295	−	2	1
ENSEASG00005010157	*HOXC5*	22	15,155,129	15,157,687	2559	−	2	1
ENSEASG00005024216	*HOXC8*	22	15,177,828	15,185,726	7899	−	3	2
ENSEASG00005010148	*HOXC9*	22	15,186,829	15,190,367	3539	−	2	1
ENSEASG00005010143	*HOXC10*	22	15,199,973	15,205,335	5363	−	2	1
ENSEASG00005010141	*HOXC11*	22	15,213,982	15,217,484	3503	−	2	1
ENSEASG00005010128	*HOXC12*	22	15,232,079	15,235,733	3655	−	3	2
ENSEASG00005032367	*HOXC13*	22	15,244,014	15,251,901	7888	−	2	1
ENSEASG00005030887	*HOXD1*	4	37,329,029	37,331,558	2530	−	2	1
ENSEASG00005022544	*HOXD3*	4	37,345,280	37,353,711	8432	−	2	1
ENSEASG00005024806	*HOXD4*	4	37,363,644	37,366,161	2518	−	2	1
ENSEASG00005033905	*HOXD8*	4	37,384,491	37,391,458	6968	−	3	2
ENSEASG00005023994	*HOXD9*	4	37,391,918	37,394,428	2511	−	2	1
ENSEASG00005022552	*HOXD10*	4	37,396,025	37,402,726	6702	−	2	1
ENSEASG00005030904	*HOXD11*	4	37,406,901	37,409,659	2759	−	3	2
ENSEASG00005022555	*HOXD12*	4	37,416,161	37,417,118	958	−	2	1
ENSEASG00005029242	*HOXD13*	4	37,421,158	37,424,066	2909	−	2	1

**Table 2:** The *HOX* gene family among donkeys, humans, horses, and cattle.

*HOX* Gene	Donkey	Human	Horses	Cattle
*HOXA*	1, 2, 3, 5, 10, 11, 13	1, 2, 3, 4, 5, 6, 7, 9, 10, 11, 13	1, 2, 3, 5, 6, 7, 9, 10, 11, 13	1, 2, 3, 4, 5, 6, 7, 9, 10, 11, 13
*HOXB*	1, 2, 3, 5, 6, 7, 8, 9, 13	1, 2, 3, 4, 5, 6, 7, 8, 9, 13	1, 2, 3, 4, 5, 8, 9, 13	1, 2, 3, 4, 5, 6, 7, 8, 9, 13
*HOXC*	4, 5, 7, 8, 9, 10, 11, 12, 13	4, 5, 6, 7, 8, 9, 10, 11, 12, 13	5, 6, 7, 8, 9, 10, 11, 12, 13	4, 5, 6, 7, 8, 9, 10, 11, 12, 13
*HOXD*	1, 2, 3, 4, 8, 9, 10, 11, 12, 13	1, 2, 3, 4, 8, 9, 10, 11, 12, 13	1, 2, 3, 4, 8, 9, 10, 11, 13	1, 2, 3, 4, 8, 9, 10, 11, 12, 13
Total	33	39	35	39

**Table 3:** Physicochemical properties of HOX gene family proteins in donkeys.

Gene ID	HOX Proteins	Number of Amino Acid	Molecular Weight	Theoretical pI	Instability Index	Aliphatic Index	Grand Average of Hydropathicity
ENSEASG00005030120	HOXA1	337	36,689.29	7.77	62.15	49.26	−0.795
ENSEASG00005033684	HOXA2	373	40,878.41	5.61	66.72	64.4	−0.653
ENSEASG00005035791	HOXA3	444	47,378.77	9.93	61.61	59.44	−0.639
ENSEASG00005034554	HOXA5	381	41,491.04	10.08	62.26	49.06	−0.837
ENSEASG00005022176	HOXA10	94	11,452.25	10.6	32.48	60.21	−1.084
ENSEASG00005022180	HOXA11	316	34,699.54	8.91	60.31	51.39	−0.728
ENSEASG00005022194	HOXA13	387	39,680.62	9.24	50.21	59.2	−0.288
ENSEASG00005000594	HOXB1	304	32,480.8	7.6	74.97	41.88	−0.809
ENSEASG00005000585	HOXB2	352	37,795.54	5.04	90.52	62.53	−0.503
ENSEASG00005000581	HOXB3	414	42,988.86	9.42	64.77	43.57	−0.774
ENSEASG00005000563	HOXB5	208	23,738.14	10.45	68.85	56.97	−0.851
ENSEASG00005000560	HOXB6	224	25,356.28	8.45	75.07	44.55	−0.894
ENSEASG00005000556	HOXB7	217	23,954.65	8.82	63.15	49.26	−0.695
ENSEASG00005024978	HOXB8	243	27,573.67	8.48	65.78	49.47	−0.97
ENSEASG00005000527	HOXB9	250	28,068.57	9.01	58.32	58.24	−0.862
ENSEASG00005025083	HOXB13	285	30,819.76	9.25	57.57	57.3	−0.604
ENSEASG00005010161	HOXC4	264	29,851.3	9.24	79.11	46.67	−1.086
ENSEASG00005010157	HOXC5	222	25,041.3	9.55	71.59	53.83	−0.859
ENSEASG00005024216	HOXC8	380	42,569.49	9.03	58.56	57.34	−0.879
ENSEASG00005010148	HOXC9	260	29,233.94	9.14	63.7	54.5	−0.781
ENSEASG00005010143	HOXC10	342	38,137.68	8.45	57.24	53.42	−0.896
ENSEASG00005010141	HOXC11	305	33,728.66	8.81	60.20	51.31	−0.745
ENSEASG00005010128	HOXC12	281	29,898.4	7.57	62.87	63.2	−0.612
ENSEASG00005032367	HOXC13	315	34,024.44	9.73	64.28	59.59	−0.609
ENSEASG00005030887	HOXD1	320	33,558.98	10.08	69.78	55.12	−0.503
ENSEASG00005022544	HOXD3	432	45,610.94	8.98	68.99	49.63	−0.708
ENSEASG00005024806	HOXD4	257	27,846.29	9.48	74.64	46.38	−0.862
ENSEASG00005033905	HOXD8	180	21,146.88	10.76	82.25	43.5	−1.518
ENSEASG00005023994	HOXD9	350	35,775.68	9.11	70.63	41.74	−0.638
ENSEASG00005022552	HOXD10	410	46,304.57	8.82	69.74	62.78	−0.659
ENSEASG00005030904	HOXD11	336	34,874.02	9.01	57.2	46.43	−0.596
ENSEASG00005022555	HOXD12	270	29,006.14	9.76	49.41	73.59	−0.409
ENSEASG00005029242	HOXD13	341	35,883.05	9.5	52.36	57.16	−0.407

**Table 4:** Secondary structure prediction and subcellular localization of HOX gene family proteins in donkeys.

HOX Proteins	Alpha Helix (%)	Extended Chain (%)	Beta Turn (%)	Random Coil (%)	Subcellular Localization
HOXA1	12.76	1.19	0.59	85.46	nucleus
HOXA2	13.14	1.61	0.54	84.72	nucleus
HOXA3	15.09	3.38	3.83	77.70	nucleus
HOXA5	13.12	1.05	1.31	84.51	nucleus
HOXA10	34.04	6.38	2.13	57.45	nucleus
HOXA11	12.03	1.27	0.95	85.76	nucleus
HOXA13	23.00	3.10	2.07	71.83	nucleus
HOXB1	14.8	2.96	0.66	81.58	nucleus
HOXB2	16.48	1.7	0.57	81.25	nucleus
HOXB3	9.66	0.97	0.48	88.89	nucleus
HOXB5	17.31	3.37	0.96	78.37	nucleus
HOXB6	19.20	2.68	0.89	77.23	nucleus
HOXB7	22.58	1.84	0.92	74.65	nucleus
HOXB8	17.70	1.65	0.82	79.84	nucleus
HOXB9	14.80	1.60	1.20	82.40	nucleus
HOXB13	17.89	2.46	2.46	77.19	nucleus
HOXC4	13.26	1.52	0.76	84.47	nucleus
HOXC5	20.72	2.70	0.90	75.68	nucleus
HOXC8	16.32	2.63	1.32	79.74	nucleus
HOXC9	17.31	2.69	1.15	78.85	nucleus
HOXC10	13.74	3.22	0.88	82.16	nucleus
HOXC11	12.46	2.30	0.98	84.26	nucleus
HOXC12	14.95	1.42	0.36	83.27	nucleus
HOXC13	16.83	1.27	1.27	80.63	nucleus
HOXD1	12.19	3.12	0.62	84.06	nucleus
HOXD3	8.80	1.16	0.46	89.58	nucleus
HOXD4	12.84	1.56	0.78	84.82	nucleus
HOXD8	18.33	2.22	1.11	78.33	nucleus
HOXD9	13.43	2.00	0.57	84.00	nucleus
HOXD10	19.02	4.39	0.98	75.61	nucleus
HOXD11	13.99	1.19	0.89	83.93	nucleus
HOXD12	17.41	3.33	2.22	77.04	nucleus
HOXD13	13.78	2.93	0.59	82.70	nucleus

**Table 5:** Interspecific homologous gene pairs of the *HOX* gene gamily in Equus asinus and Equus caballus. “==” represents the collinearity of genes in two species.

Chr	Gene Name	Gene ID		Chr	Gene ID
EA-1	*HOXA13*	ENSEASG00005022194	==	EC-11	ENSECAG00000013619
EA-1	*HOXA13*	ENSEASG00005022194	==	EC-6	ENSECAG00000024867
EA-1	*HOXA13*	ENSEASG00005022194	==	EC-4	ENSECAG00000058817
EA-1	*HOXA13*	ENSEASG00005022194	==	EC-18	ENSECAG00000020388
EA-1	*HOXA11*	ENSEASG00005022180	==	EC-11	ENSECAG00000036001
EA-1	*HOXA11*	ENSEASG00005022180	==	EC-4	ENSECAG00000012577
EA-1	*HOXA1*	ENSEASG00005030120	==	EC-18	ENSECAG00000035437
EA-1	*HOXA2*	ENSEASG00005033684	==	EC-18	ENSECAG00000022129
EA-4	*HOXD1*	ENSEASG00005030887	==	EC-4	ENSECAG00000021767
EA-4	*HOXD1*	ENSEASG00005030887	==	EC-18	ENSECAG00000035437
EA-4	*HOXD1*	ENSEASG00005030887	==	EC-11	ENSECAG00000011628
EA-4	*HOXD9*	ENSEASG00005023994	==	EC-11	ENSECAG00000036001
EA-4	*HOXD9*	ENSEASG00005023994	==	EC-18	ENSECAG00000003045
EA-4	*HOXD9*	ENSEASG00005023994	==	EC-6	ENSECAG00000024867
EA-4	*HOXD9*	ENSEASG00005023994	==	EC-4	ENSECAG00000012577
EA-4	*HOXD12*	ENSEASG00005022555	==	EC-11	ENSECAG00000013619
EA-4	*HOXD12*	ENSEASG00005022555	==	EC-4	ENSECAG00000058817
EA-4	*HOXD12*	ENSEASG00005022555	==	EC-18	ENSECAG00000038429
EA-4	*HOXD12*	ENSEASG00005022555	==	EC-6	ENSECAG00000003682
EA-4	*HOXD4*	ENSEASG00005024806	==	EC-18	ENSECAG00000021980
EA-4	*HOXD4*	ENSEASG00005024806	==	EC-6	ENSECAG00000000932
EA-4	*HOXD11*	ENSEASG00005030904	==	EC-18	ENSECAG00000020388
EA-4	*HOXD13*	ENSEASG00005029242	==	EC-4	ENSECAG00000038185
EA-13	*HOXB13*	ENSEASG00005025083	==	EC-6	ENSECAG00000024867
EA-13	*HOXB13*	ENSEASG00005025083	==	EC-4	ENSECAG00000038185
EA-13	*HOXB13*	ENSEASG00005025083	==	EC-18	ENSECAG00000020388
EA-13	*HOXB13*	ENSEASG00005025083	==	EC-11	ENSECAG00000013619
EA-13	*HOXB9*	ENSEASG00005000527	==	EC-11	ENSECAG00000036001
EA-13	*HOXB9*	ENSEASG00005000527	==	EC-4	ENSECAG00000012577
EA-13	*HOXB3*	ENSEASG00005000581	==	EC-11	ENSECAG00000011628
EA-13	*HOXB3*	ENSEASG00005000581	==	EC-18	ENSECAG00000022129
EA-13	*HOXB8*	ENSEASG00005024978	==	EC-11	ENSECAG00000028576
EA-13	*HOXB8*	ENSEASG00005024978	==	EC-6	ENSECAG00000034365
EA-13	*HOXB8*	ENSEASG00005024978	==	EC-18	ENSECAG00000020599
EA-13	*HOXB8*	ENSEASG00005024978	==	EC-4	ENSECAG00000000461
EA-13	*HOXB1*	ENSEASG00005000594	==	EC-18	ENSECAG00000035437
EA-13	*HOXB2*	ENSEASG00005000585	==	EC-4	ENSECAG00000021767
EA-13	*HOXB6*	ENSEASG00005000560	==	EC-6	ENSECAG00000000932
EA-22	*HOXC4*	ENSEASG00005010161	==	EC-11	ENSECAG00000011628
EA-22	*HOXC4*	ENSEASG00005010161	==	EC-18	ENSECAG00000021980
EA-22	*HOXC4*	ENSEASG00005010161	==	EC-4	ENSECAG00000021767
EA-22	*HOXC4*	ENSEASG00005010161	==	EC-6	ENSECAG00000003682
EA-22	*HOXC5*	ENSEASG00005010157	==	EC-11	ENSECAG00000002707
EA-22	*HOXC5*	ENSEASG00005010157	==	EC-6	ENSECAG00000000932
EA-22	*HOXC8*	ENSEASG00005024216	==	EC-11	ENSECAG00000036001
EA-22	*HOXC8*	ENSEASG00005024216	==	EC-18	ENSECAG00000021845
EA-22	*HOXC8*	ENSEASG00005024216	==	EC-4	ENSECAG00000000575
EA-22	*HOXC8*	ENSEASG00005024216	==	EC-6	ENSECAG00000044561
EA-22	*HOXC13*	ENSEASG00005032367	==	EC-11	ENSECAG00000013619
EA-22	*HOXC13*	ENSEASG00005032367	==	EC-4	ENSECAG00000058817
EA-22	*HOXC13*	ENSEASG00005032367	==	EC-6	ENSECAG00000024867
EA-22	*HOXC9*	ENSEASG00005010148	==	EC-18	ENSECAG00000020388
EA-22	*HOXC9*	ENSEASG00005010148	==	EC-6	ENSECAG00000024893
EA-22	*HOXC9*	ENSEASG00005010148	==	EC-4	ENSECAG00000054482
EA-22	*HOXC11*	ENSEASG00005010141	==	EC-6	ENSECAG00000024900

**Table 6:** Ka/Ks analysis of *HOX* gene family members in donkey.

Gene Pairs	Ka	Ks	Ka/Ks	Effective Len
*HOXA13-HOXB13*	0.376840	2.112613076	0.178376136	834
*HOXA13-HOXC9*	0.688802	1.233295288	0.558505336	567
*HOXA13-HOXD10*	0.956989	1.924073638	0.497376399	936
*HOXA5-HOXB5*	0.412243	0.964873614	0.427251108	567
*HOXA5-HOXC5*	0.479916	1.722981027	0.278538069	474
*HOXA1-HOXB2*	0.713682	2.508548077	0.284500119	651
*HOXD1-HOXB3*	0.653653	1.224899147	0.533638036	687
*HOXD1-HOXC4*	0.661190	2.072512815	0.319028356	693
*HOXD3-HOXB6*	0.655668	1.488153025	0.440591488	567
*HOXD9-HOXA11*	0.707586	1.08587469	0.651627748	804
*HOXD9-HOXB9*	0.374105	1.393116473	0.268537853	738
*HOXD9-HOXC9*	0.284749	1.246390836	0.228458808	738
*HOXD10-HOXA13*	0.956989	1.924073638	0.497376399	936
*HOXB8-HOXC4*	0.570391	2.354667195	0.242238421	621
*HOXB8-HOXD4*	0.592094	1.046701241	0.565676685	636
*HOXB5-HOXA5*	0.412243	0.964873614	0.427251108	567
*HOXB6-HOXD3*	0.655668	1.488153025	0.440591488	567
*HOXB13-HOXA13*	0.376840	2.112613076	0.178376136	834
*HOXB13-HOXC9*	0.722459	1.040290463	0.694478247	720
*HOXB13-HOXD11*	0.703023	1.111068647	0.632744988	717
*HOXB2-HOXA1*	0.713682	2.508548077	0.284500119	651
*HOXC12-HOXD11*	0.683748	1.135400167	0.602208582	702
*HOXC4-HOXB8*	0.570391	2.354667195	0.242238421	621
*HOXC4-HOXD1*	0.661190	2.072512815	0.319028356	693
*HOXC5-HOXA5*	0.479916	1.722981027	0.278538069	474
*HOXC5-HOXB9*	0.733470	1.998865676	0.366943172	615
*HOXC9-HOXA13*	0.738721	1.055208462	0.700071555	612
*HOXC9-HOXB13*	0.722459	1.040290463	0.694478247	720
*HOXC9-HOXD9*	0.284749	1.246390836	0.228458808	738

## Data Availability

The original contributions presented in this study are included in the article/Supplementary Materials. Further inquiries can be directed to the corresponding author.

## Supplementary Materials

The following supporting information can be downloaded at: https://www.mdpi.com/article/10.3390/ijms27010038/s1.

## References

[1] Mallo M., Alonso C.R. (2013). The regulation of Hox gene expression during animal development. Development.

[2] Mallo M., Wellik D.M., Deschamps J. (2010). Hox genes and regional patterning of the vertebrate body plan. Dev. Biol..

[3] Hubert K.A., Wellik D.M. (2023). Hox genes in development and beyond. Development.

[4] Singh N.P., Krumlauf R. (2022). Diversification and functional evolution of HOX proteins. Front. Cell Dev. Biol..

[5] Darbellay F., Bochaton C., Lopez-Delisle L., Mascrez B., Tschopp P., Delpretti S., Zakany J., Duboule D. (2019). The constrained architecture of mammalian Hox gene clusters. Proc. Natl. Acad. Sci. USA.

[6] Gaunt S.J. (2022). Seeking Sense in the Hox Gene Cluster. J. Dev. Biol..

[7] van den Akker E., Fromental-Ramain C., de Graaff W., Le Mouellic H., Brûlet P., Chambon P., Deschamps J. (2001). Axial skeletal patterning in mice lacking all paralogous group 8 Hox genes. Development.

[8] Wellik D.M., Capecchi M.R. (2003). Hox10 and Hox11 Genes Are Required to Globally Pattern the Mammalian Skeleton. Science.

[9] Horan G.S., Kovàcs E.N., Behringer R.R., Featherstone M.S. (1995). Mutations in Paralogous Hox Genes Result in Overlapping Homeotic Transformations of the Axial Skeleton: Evidence for Unique and Redundant Function. Dev. Biol..

[10] Mcintyre D.C., Rakshit S., Yallowitz A.R., Loken L., Jeannotte L., Capecchi M.R., Wellik D.M. (2007). Hox patterning of the vertebrate rib cage. Development.

[11] Zhang W., Zhang M., Sun Y., Liu S. (2024). Factors affecting the quality and nutritional value of donkey meat: A comprehensive review. Front. Vet. Sci..

[12] Zhang J., Wu M., Ma Z., Zhang Y., Cao H. (2022). Species-specific identification of donkey-hide gelatin and its adulterants using marker peptides. PLoS ONE.

[13] Liu Z., Gao Q., Wang T., Chai W., Zhan Y., Akhtar F., Zhang Z., Li Y., Shi X., Wang C. (2022). Multi-Thoracolumbar Variations and NR6A1 Gene Polymorphisms Potentially Associated with Body Size and Carcass Traits of Dezhou Donkey. Animals.

[14] Zhao Y., Zhang J., Sun Z., Tang Y., Wu Y. (2021). Genome-Wide Identification and Analysis of the Polycomb Group Family in Medicago truncatula. Int. J. Mol. Sci..

[15] Hahn M.W., Han M.V., Han S. (2007). Gene Family Evolution across 12 Drosophila Genomes. PLoS Genet..

[16] Bayramov A.V., Ermakova G.V., Kuchryavyy A.V., Zaraisky A.G. (2021). Genome duplications as the basis of vertebrates’ evolutionary success. Russ. J. Dev. Biol..

[17] Aase-Remedios M.E., Ferrier D.E.K. (2021). Improved understanding of the role of gene and genome duplications in chordate evolution with new genome and transcriptome sequences. Front. Ecol. Evol..

[18] Hoegg S., Meyer A. (2005). Hox clusters as models for vertebrate genome evolution. Trends Genet..

[19] Mohanta T.K., Khan A., Hashem A., Abd Allah E.F., Al-Harrasi A. (2019). The molecular mass and isoelectric point of plant proteomes. BMC Genom..

[20] De S., Sur K., Dasgupta S. (2005). Characterization of the nonregular regions of proteins by a contortion index. Biopolymers.

[21] Uversky V.N. (2010). The Mysterious Unfoldome: Structureless, Underappreciated, Yet Vital Part of Any Given Proteome. BioMed Res. Int..

[22] Stadhouders R., Filion G.J., Graf T. (2019). Transcription factors and 3D genome conformation in cell-fate decisions. Nature.

[23] Suter D.M. (2020). Transcription factors and DNA play hide and seek. Trends Cell Biol..

[24] Mizuno H., Katagiri S., Kanamori H., Mukai Y., Sasaki T., Matsumoto T., Wu J. (2020). Evolutionary dynamics and impacts of chromosome regions carrying R-gene clusters in rice. Sci. Rep..

[25] Han Y., Luthe D. (2021). Identification and evolution analysis of the JAZ gene family in maize. BMC Genom..

[26] Brannan E.O., Hartley G.A., O’Neill R.J. (2024). Mechanisms of Rapid Karyotype Evolution in Mammals. Genes.

[27] Song J.Y., Pineault K.M., Dones J.M., Raines R.T., Wellik D.M. (2020). Hox genes maintain critical roles in the adult skeleton. Proc. Natl. Acad. Sci. USA.

[28] Shi X., Li Y., Wang T., Ren W., Huang B., Wang X., Liu Z., Liang H., Kou X., Chen Y. (2022). Association of HOXC8 Genetic Polymorphisms with Multi-Vertebral Number and Carcass Weight in Dezhou Donkey. Genes.

[29] Gonçalves C.S., Le Boiteux E., Arnaud P., Costa B.M. (2020). HOX gene cluster (de)regulation in brain: From neurodevelopment to malignant glial tumours. Cell. Mol. Life Sci..

[30] Chan K., Li X. (2021). Current Epigenetic Insights in Kidney Development. Genes.

[31] Yang M., Li Q., Zhang F. (2010). HOXgenes in the skin. Chin. Med. J..

[32] Awgulewitsch A. (2003). Hox in hair growth and development. Sci. Nat..

[33] Wang X., Ren W., Peng Y., Khan M.Z., Liang H., Zhang Y., Liu X., Chen Y., Kou X., Wang L. (2024). Elucidating the Role of Transcriptomic Networks and DNA Methylation in Collagen Deposition of Dezhou Donkey Skin. Animals.

[34] Poliacikova G., Maurel-Zaffran C., Graba Y., Saurin A.J. (2021). Hox Proteins in the Regulation of Muscle Development. Front. Cell Dev. Biol..

[35] Tian W., Zhao L., Wang J., Suo P., Wang J., Cheng L., Cheng Z., Jia J., Kan S., Wang B. (2012). Association analysis between HOXD9 genes and the development of developmental dysplasia of the hip in Chinese female Han population. BMC Musculoskelet. Disord..

[36] Clark D.L., Boler D.D., Kutzler L.W., Jones K.A., McKeith F.K., Killefer J., Carr T.R., Dilger A.C. (2011). Muscle gene expression associated with increased marbling in beef cattle. Anim. Biotechnol..

[37] González-Prendes R., Quintanilla R., Mármol-Sánchez E., Pena R.N., Ballester M., Cardoso T.F., Manunza A., Casellas J., Cánovas Á., Díaz I. (2019). Comparing the mRNA expression profile and the genetic determinism of intramuscular fat traits in the porcine gluteus medius and longissimus dorsi muscles. BMC Genom..

[38] Fischbach N.A., Rozenfeld S., Shen W., Fong S., Chrobak D., Ginzinger D., Kogan S.C., Radhakrishnan A., Le Beau M.M., Largman C. (2005). HOXB6 overexpression in murine bone marrow immortalizes a myelomonocytic precursor in vitro and causes hematopoietic stem cell expansion and acute myeloid leukemia in vivo. Blood.

[39] Lin B., Zhou X., Jiang D., Shen X., Ouyang H., Li W., Xu D., Fang L., Tian Y., Li X. (2023). Comparative transcriptomic analysis reveals candidate genes for seasonal breeding in the male Lion-Head goose. Br. Poult. Sci..

[40] Topaloğlu U., Akbalik M.E., Sağsöz H. (2021). Immunolocalization of some HOX proteins in immature and mature feline testes. Anat. Histol. Embryol..

[41] Bomgardner D., Hinton B.T., Turner T.T. (2001). Hox Transcription Factors May Play a Role in Regulating Segmental Function of the Adult Epididymis. J. Androl..

[42] Mandeville I., Aubin J., Leblanc M., Lalancette-Hébert M., Janelle M., Tremblay G.M., Jeannotte L. (2006). Impact of the Loss of Hoxa5 Function on Lung Alveogenesis. Am. J. Pathol..

