# Supplementary material for: Genome-Wide Characterization of the HOX Gene Family: Evolution and Expression Patterns in Donkey
Source: Int J Mol Sci. 2025 Dec 19;27(1):38. doi: 10.3390/ijms27010038 (PMC12785281; doi:10.3390/ijms27010038)
Supplement: Supplementary file 1 [file ijms-27-00038-s001.zip › ijms-3972002-Supplementary Materials S1.pdf]

Supplementary Materials S1. Tissue sample information in RNA-seq and average FPKM values for HOX gene identified in 13 tissue transcriptomes.

Gene name	Heart	Kidney	Lung	Brain	Spleen
HOXA1	0.647313333	2.088201333	0.698969667	0	0.325215
HOXA2	0.761944	2.217973333	3.563535333	0.036636667	0.065547333
HOXA3	2.002402667	10.28181233	9.784955333	0	0.530937667
HOXA5	2.711632667	16.79572	32.063034	0	0.784659
HOXA10	0	13.24926133	0.039780333	0	0.026007
HOXA11	0	1.038580333	0	0	0
HOXA13	0.28609	0.22453	0	0	0
HOXB1	0	0	0	0	0
HOXB2	8.612054333	19.74387633	5.764605	0	4.986686667
HOXB3	6.686955667	12.80876233	5.15174	0	3.289214333
HOXB5	0.128832333	17.493927	2.457529333	0.111264	0.045445
HOXB6	0.513059	47.397644	3.858852333	0.105407	0.330010333
HOXB7	0	25.605041	2.268056667	0	0.472550667
HOXB8	0	17.79435	0.263558	0	0
HOXB9	0	2.541189333	0	0	0
HOXB13	0	0	0.070668667	0	0.023872
HOXC4	0.225617333	3.211708667	1.432304667	0	1.018380333
HOXC5	0	0.524474	0.215306667	0	0
HOXC8	0	1.623558	0	0	0
HOXC9	0	14.18160433	0	0	0.086050333
HOXC10	0	4.669462333	0	0	0
HOXC11	0	0.380527	0	0	0
HOXC12	0	0	0	0	0
HOXC13	0	0	0	0	0
HOXD1	0.033349	5.748542	0	0.780007667	0
HOXD3	0.048166	12.42720267	0.044408	0.032742667	0.028981
HOXD4	0.440817333	6.673627333	0.058648	0	0.206295
HOXD8	0.888241333	42.46442033	0.278398333	0	0.525007667
HOXD9	1.172928667	18.482912	0.271094667	0.033947333	0.589206333
HOXD10	0.169947	8.062419333	0.062243	0	0.092991333
HOXD11	0	4.010638333	0	0	0
HOXD12	0	0.036702	0	0	0
HOXD13	0	0.037323667	0	0	0.018195

Stomach	Blood	Liver	Cecum	Skin	Muscle
0.360025	0.021284333	0.159749	0.825183	0.931006333	0.202707333
0.799867333	0	0.496093	2.777651	3.034207667	0.716989333
1.987229667	0.202604667	0.419811	5.776348	3.983124333	1.441101333
3.482890667	0.310627	0.989869	36.39665	8.903194667	2.714035667
0.055731	0.042276	0	19.81726	1.001045333	2.665654667
0	0	0	4.676607	0.226499667	0
0.026642333	0	0	0.280244	0.128680333	0
0	0	0	0	0	0
3.975814667	0	1.869137	7.18335	3.252305333	1.172634333
5.314691333	0.029088333	1.656592	16.57484	4.443269	1.579760333
3.053846333	0	0.199666	35.68435	1.400936	0.447924667
8.041178	1.556680667	0.090503	115.9929	3.748271333	1.314227667
8.289594333	0.338839333	0.049014	14.0504	8.869353333	2.777908
4.465109667	0	0	3.418795	0.541387	0.045763
0.07815	0	0	13.35569	0.249707333	0.028986667
0	0	0	0.1051	0	0
5.307248667	0.604181	0.118435	1.608028	2.764982	1.125074667
2.527327333	0	0	1.682418	1.315301333	0.457846667
0.80723	0	0	2.31448	6.130948	9.550138333
0.086177667	0.029031333	1.109124	1.953715	4.810334333	11.85188633
0	0	0.08669	0.9698	1.612282	8.624063
0	0	0	0.347794	1.134688	0.032494667
0	0	0	0	1.449083667	0
0	0	0	0	15.30881767	0
0.070164667	0	0	0.115868	0.229384333	2.468783667
0.182972333	0	0	2.051733	1.354684	0.421372667
0.455277333	0	0.005405	2.862658	1.554174667	0.892223333
0.447444333	0	0.058669	4.314102	3.545918333	13.782642
1.029716667	0.075332667	0	6.894987	5.316023	15.45176167
0.117682667	0	0	9.875479	5.141509333	0.083362333
0	0.18391	0	7.636022	2.121162333	0
0	0	0	0.062764	0	0
0	0.07534	0	0.273491	1.161580333	0

Testis	Epididymis
0	1.887661333
0.029726	11.159231
0.844177667	31.67597767
0.187801	52.58420533
0.270016667	22.608006
0.093656667	8.301021667
0.389391	0.065647667
0	0
0.399819	26.54107233
2.004386	38.00034833
0.017519333	32.21572167
0	220.4862467
0.035091667	52.36494967
0.024972667	69.88591767
0.025825333	5.763436
0	0
0.819439667	4.935181333
0.212629	1.250137333
0.087018	1.439279
0.935691667	2.425672
0.070932667	1.736586333
0.031603667	1.593088
0.474712	0
0.04147	0
0.533716333	9.681821333
0.298057667	25.37698567
0.580652667	18.379897
34.219413	77.16043767
1.178791667	27.68185233
0.208916667	10.17832333
0.171863333	3.252469
0.366374667	0
0.577463667	0.104023
