# Supplementary figures and images for: Genome-Wide Characterization of the HOX Gene Family: Evolution and Expression Patterns in Donkey
Source: Int J Mol Sci. 2025 Dec 19;27(1):38. doi: 10.3390/ijms27010038 (PMC12785281; doi:10.3390/ijms27010038)

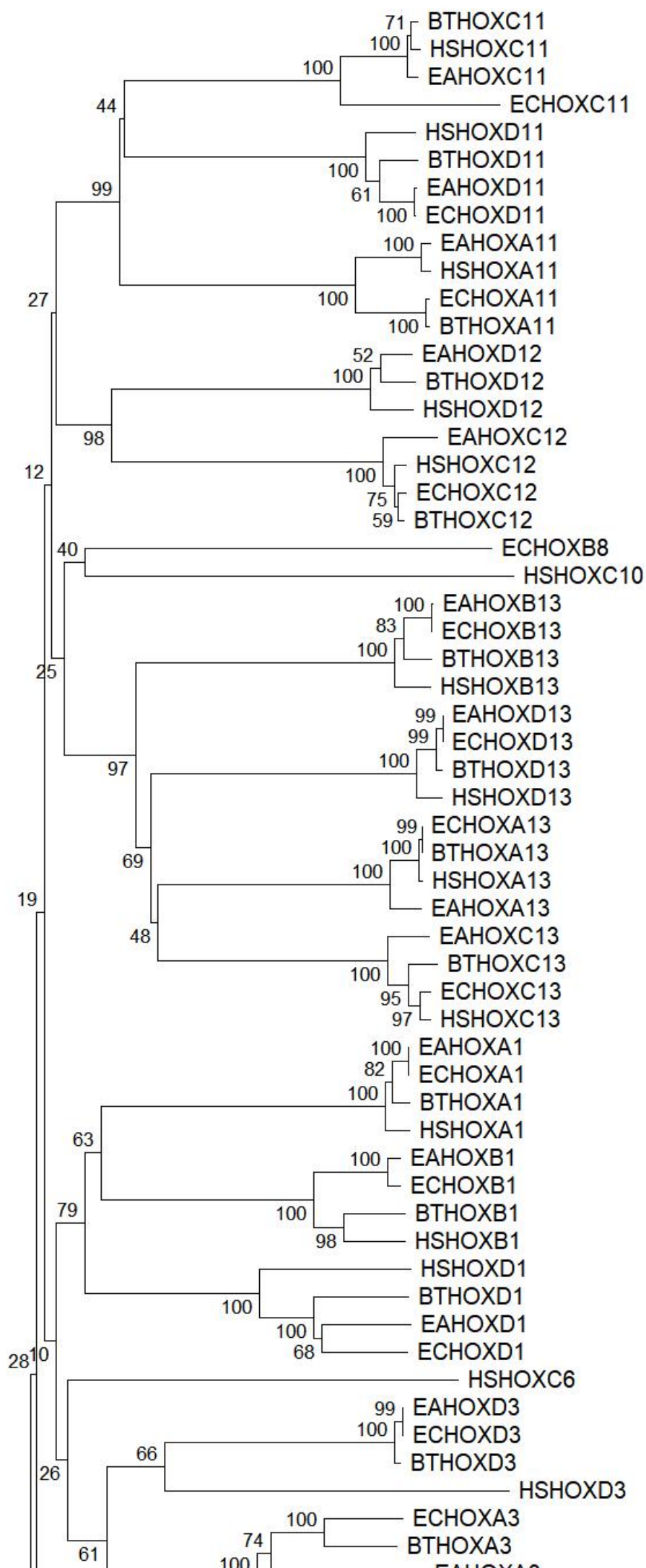

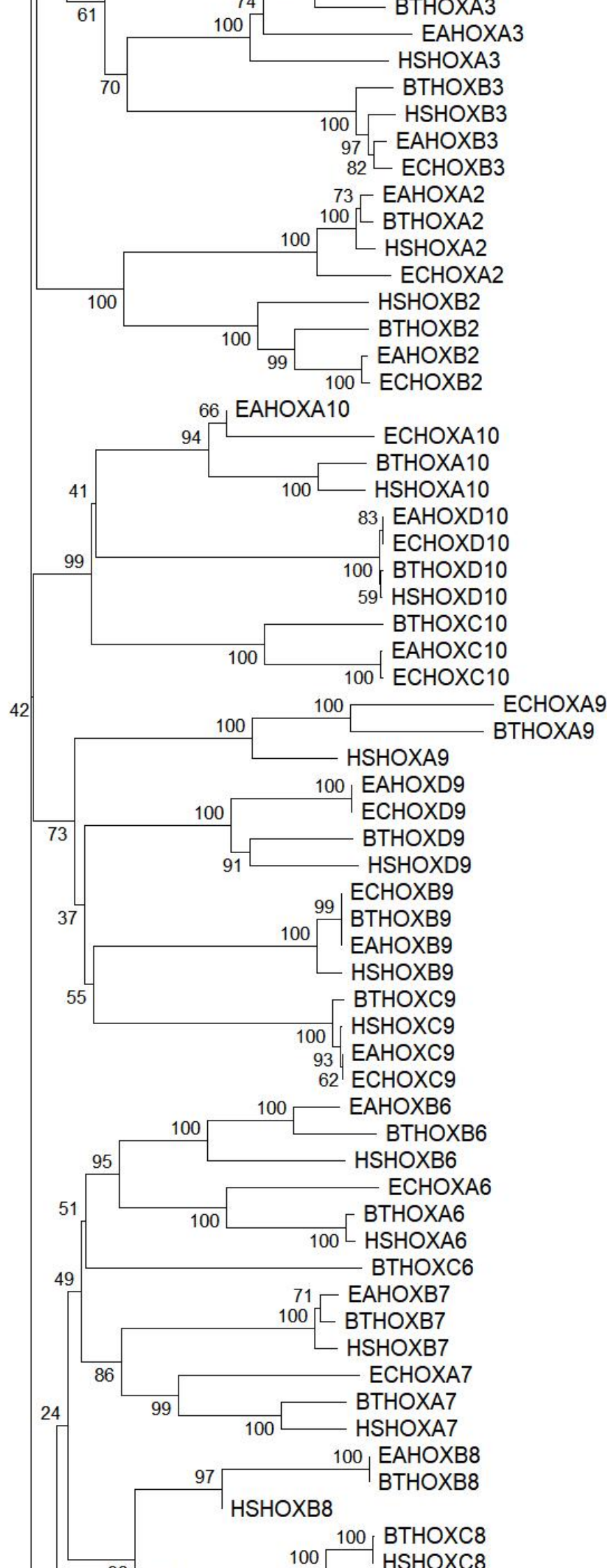

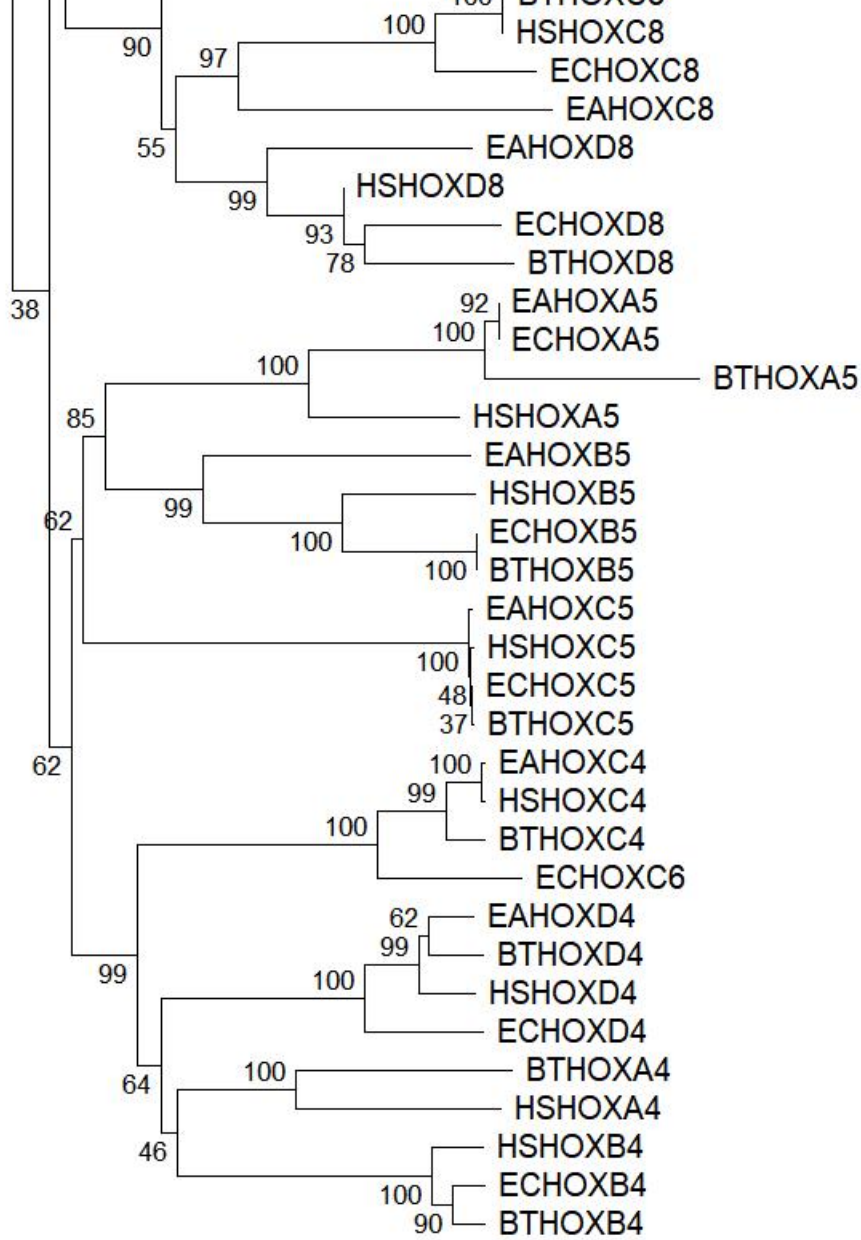

**Supplementary Materials S2.** The phylogenetic tree.

Supplement: Supplementary file 1 [file ijms-27-00038-s001.zip › ijms-3972002-Supplementary Materials S2.pdf]
